# Development of pristine and Au-decorated Bi_2_O_3_/Bi_2_WO_6_ nanocomposites for supercapacitor electrodes†

**DOI:** 10.1039/c9ra06112f

**Published:** 2019-10-11

**Authors:** Gorkshnath H. Gote, Mansi Pathak, Mahendra A. More, Dattatray J. Late, Chandra Sekhar Rout

**Affiliations:** Centre for Advanced Studies in Materials Science and Condensed Matter Physics, Department of Physics, Savitribai Phule Pune University Pune Maharashtra 411007 India mam@physics.unipune.ac.in; Physical and Materials Chemistry Division, CSIR-National Chemical Laboratory Dr Homi Bhabha Road, Pashan Pune Maharashtra 411008 India datta099@gmail.com dj.late@ncl.res.in; Centre for Nano and Material Sciences Jain Global Campus, Jakkasandra, Ramanagaram Bangalore-562112 India r.chandrasekhar@jainuniversity.ac.in

## Abstract

Pristine and Au-decorated Bi_2_O_3_/Bi_2_WO_6_ nanocomposites were synthesized *via* a facile hydrothermal method. Characterization techniques such as XRD, FESEM, HRTEM and XPS were used to explore the structural, morphological and electronic properties. Furthermore, electrochemical characterizations including cyclic voltammetry (CV), the galvanostatic charge–discharge (GCD) method, and electrochemical impedance spectroscopy (EIS) were performed to investigate the supercapacitance behaviour of the synthesized materials. Interestingly, the Au-decorated Bi_2_O_3_/Bi_2_WO_6_ nanocomposite showed a higher capacitance of 495.05 F g^−1^ (1 M aqueous KOH electrolyte) with improved cycling stability (99.26%) over 2000 cycles, measured at a current density of 1 A g^−1^, when compared to the pristine Bi_2_O_3_/Bi_2_WO_6_ composite (capacitance of 148.81 F g^−1^ and good cycling stability (95.99%) over 2000 cycles at a current density of 1 A g^−1^). The results clearly reveal that the decoration of the Bi_2_O_3_/Bi_2_WO_6_ composite with Au nanoparticles enhances its supercapacitance behaviour, which can be attributed to an increase in electrical conductivity, good electrical contact between the electrode and electrolyte, and an increase in effective area. The Au-decorated Bi_2_O_3_/Bi_2_WO_6_ nanocomposite can be considered as an electrode material for supercapacitor application.

## Abstract 2

Pristine and Au-decorated Bi_2_O_3_/Bi_2_WO_6_ nanocomposites were synthesized *via* a facile hydrothermal method, and find its application in supercapacitor.

## Graphical abstract

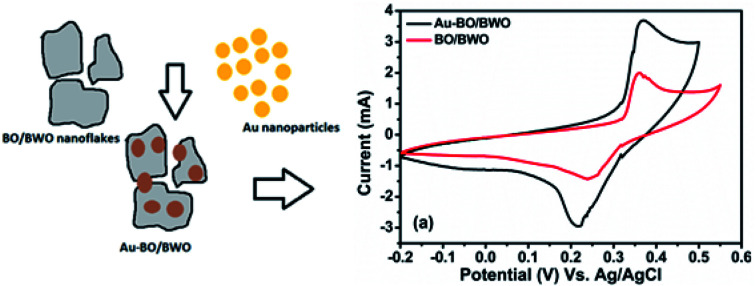

## Introduction

Due to the increasing demand for energy, the fields of energy storage and conservation have emerged as front-line research areas. In the quest for sustainable, high-performance and efficient technology, nanostructured materials and their composites have attracted a great deal of interest as building blocks. In the context of energy storage and supply devices, supercapacitors have gained significant attention as they exhibit higher energy density than conventional capacitors and higher power density than batteries.^1–3^ Various metal-oxides such as MoS_2_,^4^ MnO_2_,^5^ WO_3_,^6^ Bi_2_O_3_,^7–9^*etc.* have been explored as pseudocapacitive supercapacitor electrode materials. Bi_2_O_3_ has been studied widely as a material for supercapacitor electrodes because of its oxide-ion conductivity, band-gap and high electrochemical stability.^7–11^ Due to its ferroelectric and oxide-ion-conductive properties, Bi_2_WO_6_ exhibits good physical and chemical properties, and luminescence activity.^12^ It belongs to the Aurivillius phase crystal family in which the perovskite layers of (WO_4_)_2_ lie between (Bi_2_O_2_)^2+^ layers.^12–14^ The Bi_2_WO_6_ composite with Bi_2_O_3_ possesses a small crystallite size and a very good visible-light response, thereby exhibiting enhanced photocatalytic activity.^15^ Au-decorated Bi_2_WO_6_ shows higher photocatalytic activity than the pristine Bi_2_WO_6_, owing to the surface plasmon effect of the Au nanoparticles, and electron trapping activity.^16,17^ Bi_2_O_3_ is a p-type semiconductor (band-gap = 2.8 eV) and it is also being explored as a photocatalytic material for its ability to oxidize water and generate reactive species, which can initiate the oxidation reaction.^18^ Various composites such as Bi_2_O_3_/TiO_2_,^19^ Bi_2_O_3_/NiO^20^ and BiOCl/Bi_2_O_3_,^21^ obtained by photosensitization of Bi_2_O_3_ in visible light, have shown improved photocatalytic activities, due to the efficient separation of photo-excited electrons and holes. Moreover, one of the advantages of nanometre sized semiconductor materials is that they possess higher catalytic activities.

In this work, we report the electrochemical behaviour of pristine Bi_2_O_3_/Bi_2_WO_6_ (abbreviated as BO/BWO) and Au-nanoparticle-decorated BO/BWO nanocomposites (Au–BO/BWO) as high-performance supercapacitor electrodes. The composites were synthesized *via* a facile hydrothermal route and the electrochemical performances of the as-prepared BO/BWO and Au–BO/BWO nanocomposites were tested using three electrodes set up in 1 M KOH electrolyte. A specific capacitance of 148.81 F g^−1^ and good cycling stability over 2000 cycles (at the current density of 1 A g^−1^) with 95.99% of capacitance retention were obtained for the pristine BO/BWO. Furthermore, the Au–BO/BWO nanocomposite showed an enhanced capacitance of 495.05 F g^−1^ and very good cycling stability over 2000 cycles (at the current density of 1 A g^−1^). Thus, the Au–BO/BWO nanocomposite can be considered as a potential candidate for application in supercapacitor electrodes.

## Experimental section

### Synthesis of BO/BWO

The BO/BWO composite was synthesized *via* the hydrothermal route with slight modifications to the literature method.^22^ All the precursors were of analytical grade and were used without further purification. Na_2_WO_6_·H_2_O (1.21 mmol) was dissolved in 20 ml of distilled water and stirred for 30 min. In another beaker, 1.85 mmol of Bi(NO_3_)_3_·5H_2_O was dissolved in 40 ml of distilled water and stirred for 30 min. Both of the solutions were mixed together and 2.19 mmol of CTAB (C_19_H_42_BrN) was added slowly. The resultant solution was ultra-sonicated for 10 min and then transferred into an 80 ml Teflon-lined stainless steel autoclave. The reaction was left to proceed for 24 h at 160 °C and then the system was allowed to cool down to room temperature. The resultant product was harvested by filtering. The precipitate was washed thoroughly with distilled water and ethanol, and centrifuged at 4000 rpm. The product was vacuum dried overnight and used for further characterization.

### Synthesis of Au–BO/BWO

The Au–BO/BWO composite was prepared following a homogeneous deposition–precipitation (HDP) route.^23^ In a typical synthesis experiment, 30 mg of Au precursor (HAuCl_4_·3H_2_O) was dissolved into 80 ml distilled water. Then, 0.9 g of urea was dissolved into the solution and it was stirred for 1 h. Next, 150 mg of BO/BWO powder was dispersed into the above solution and the mixture was sonicated for 30 min. The temperature of the resultant mixture was increased gradually to 90 °C and maintained at that temperature for 4 h. The mixture was then cooled down naturally to room temperature, and the residue was collected by filtration and washed several times with distilled water. The resultant product was dried at 80 °C overnight and calcinated at 300 °C for 4 h in air so as to remove impurities and unreacted species.

### Structural characterization

The crystallographic data for BO/BWO and Au–BO/BWO were obtained using a Rigaku MicroMax 007 HF X-ray generator (Cu Kα, *λ* = 1.54 Å) operated at 40 kV and 30 mA. For morphological analyses, field emission scanning electron microscopy (FESEM, FEI Nova NanoSEM 450 with accelerating voltage 18 kV) was used. Detailed structural and morphological analyses were carried out using transmission electron microscopy (TEM, JEOL JEM F200). X-ray photoelectron spectroscopy was performed on a Thermo Kα+ spectrometer (Al Kα X-rays with energy ∼ 1486.6 eV). The electrochemical characteristics of the prepared electrodes, including CV, GCD, and impedance spectroscopy measurements, were obtained using a PG262A Potentiostat/Galvanostat (PGSTAT) (Techno Science Ltd. Bangalore, India).

### Electrochemical measurements

The electrochemical performance was studied using 1 M aqueous KOH as the electrolyte and a typical three-electrode cell, wherein Pt wire, Ag/AgCl (in 1 M KCl) and an as-prepared material sample were used as the counter, reference, and working electrodes, respectively. In another set of experiments, a two-electrode set-up (Swagelok type cell) was used and measurements were taken in 6 M KOH electrolyte. The electrodes were soaked in the electrolyte for 3 h and a piece of Whatman paper was used as a separator. The working electrodes were fabricated on Ni foam (diameter 1 cm for 2 electrode measurements) by a simple drop-cast method. For each material (BO/BWO and Au–BO/BWO), 1 mg was dispersed in a mixture of Nafion and ethanol, and sonicated for 10 min. The slurry was drop-cast on Ni foam and dried in a vacuum at 60 °C. Then the substrate was pellet pressed with a pressure of 5 tons using a hydraulic pellet press machine, and the electrodes were ready for measurements.

## Results and discussions

**Fig. 1:**
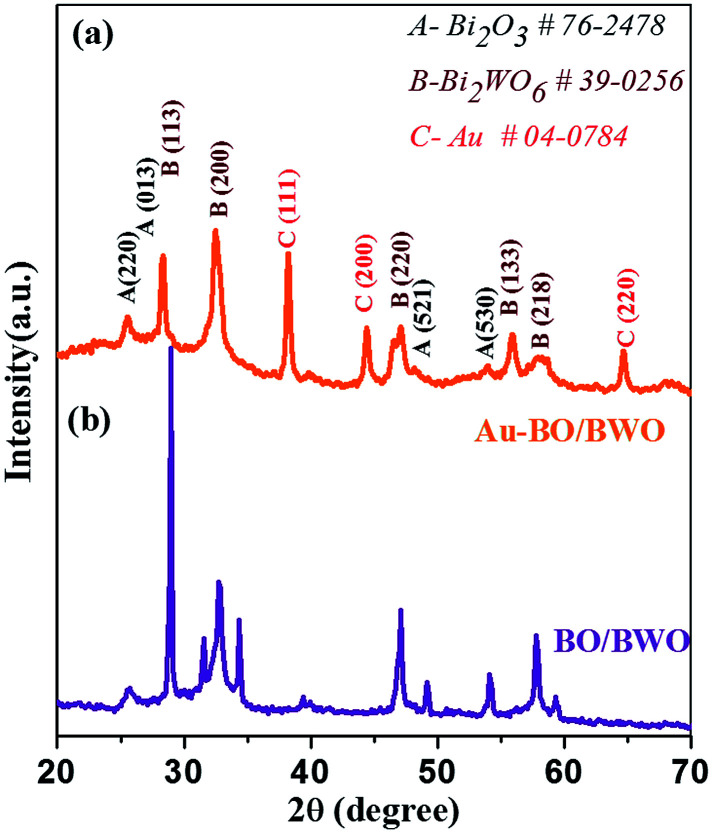
XRD pattern of the (a) Au–BO/BWO and (b) BO/BWO nanocomposites.

Fig. 1 displays the X-Ray Diffraction (X-RD) patterns of the as-synthesized BO/BWO and Au–BO/BWO samples. The well-defined peaks indicate the formation of crystalline phases in both of the samples. The observed diffraction peaks can be assigned to the cubic phase of Bi_2_O_3_ (*a* = *b* = *c* = 5.450 Å) (JCPDS no. 76-2478)^15^ and the orthorhombic phase of Bi_2_WO_6_ (*a* = 5.457 Å, *b* = 16.435 Å, *c* = 5.438 Å) (JCPDS no. 39-0256).^15^ The XRD pattern of Au–BO/BWO depicts sharp peaks due to the crystalline phase of Au metal (JCPDS no. 04-0784) in addition to the characteristic diffraction signatures of both cubic Bi_2_O_3_ and orthorhombic Bi_2_WO_6_.^17^ The observed diffraction peaks due to Au do not exhibit significant shifts with respect to their standard 2*θ* values, which is indicative of the presence of Au nanoparticles decorating the BO/BWO composite (as confirmed by the FESEM and TEM analyses).^17^

**Fig. 2:**
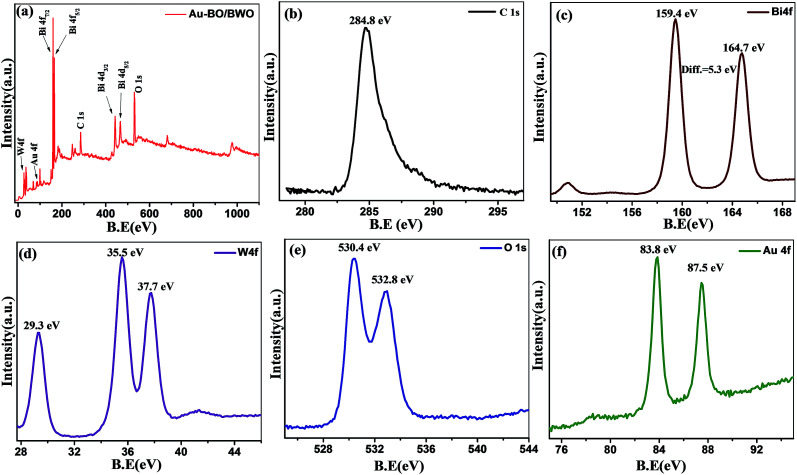
(a) Survey spectrum of the Au–BO/BWO nanocomposite; (b) C 1s, (c) Bi 4f, (d) W 4f, (e) O 1s and (f) Au 4f spectra of the Au–BO/BWO nanocomposite.

The chemical properties of the Au–BO/BWO nanocomposite were obtained from the XPS analysis. The survey scan (Fig. 2(a)) depicts characteristic signatures due to Au, Bi, O, W, and C. The peak observed at 284.8 eV corresponding to C 1s (Fig. 2(b)) is due to adventitious carbon/hydrocarbon species physisorbed on the sample surface as it is exposed to the air.^19^ The resolved spectrum of the Bi 4f level (Fig. 2(c)) shows peaks at binding energies of ∼164.7 and 159.4 eV, which can be assigned to Bi 4f_5/2_ and Bi 4f_7/2,_ respectively, implying that the Bi species exists in the trivalent oxidation state. A careful observation reveals that, in comparison to the binding energies of Bi^3+^ in the pure Bi_2_WO_6_ and Bi_2_O_3_ phases, the observed peaks show slight shifts in their positions.^10,16,19^ This is indicative of the formation of a composite, rather than the presence of a mixture of individual phases. Thus, the XPS analysis confirms the formation of a BO/BWO composite under the prevailing experimental conditions. The resolved XPS spectrum of the W 4f level is given in Fig. 2(d). It exhibits two peaks at 35.5 and 37.7 eV, representative of the 4f_7/2_ and 4f_5/2_ states, and corresponding to an oxidation number of six for W (*i.e.* W^6+^).^24^ The peaks observed at binding energies of ∼530.4 and 532.8 eV (Fig. 2(e)) in the O 1s level spectrum indicate absorbed oxygen in the crystal lattice, and can be assigned to Bi–O and W–O bonding in the composite.^10,16^ The resolved spectrum of the Au 4f level (Fig. 2(f)) shows peaks at binding energies of ∼83.8 eV and 87.5 eV, associated with the Au 4f_7/2_ and 4f_5/2_ states, respectively, and suggests the presence of metallic Au^0^ species in the sample. Thus, the XPS results clearly indicate the presence of metallic phase Au on the surface of the BO/BWO composite.^16,17^

**Fig. 3:**
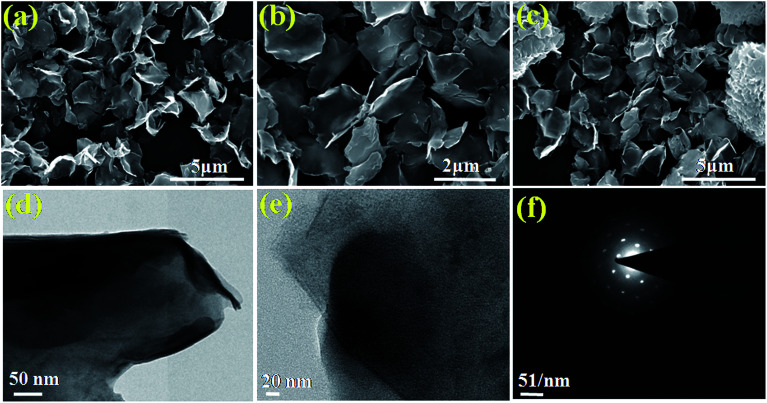
(a–c) FESEM images of BO/BWO at different magnifications. (d and e) HRTEM images and (f) SAED pattern of BO/BWO.

**Fig. 4:**
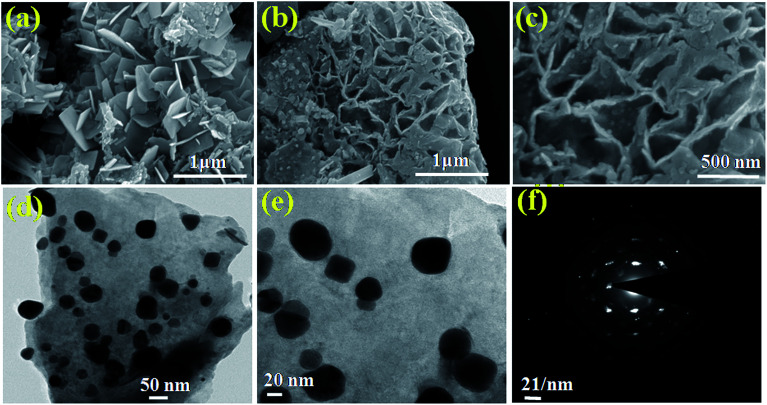
(a–c) FESEM images of Au–BO/BWO at different magnifications. (d and e) HRTEM images and (f) SAED pattern of Au–BO/BWO.

The morphological analysis of the as-synthesized products was conducted using FESEM, followed by TEM (high resolution TEM (HRTEM) and selected area electron diffraction (SAED)) so as to gain a better understanding. The FESEM images of the pristine BO/BWO composite recorded at different magnifications (Fig. 3(a–c)) reveal that the surface is characterised by randomly oriented micron-sized flake-like structures of nanometric thickness. Fig. 3(d and e) and Fig. 3(f) depict high resolution TEM images and the SAED pattern, respectively, which reveal that the flakes are ultrathin and single crystalline in nature.^18^ The morphology of the Au–BO/BWO composite, as seen from the FESEM images (Fig. 4(a–c)) is identical to that of the pristine BO/BWO composite. A careful observation of the FESEM image recorded at higher magnification reveals the presence of tiny particles (of nanometric dimensions) on the flakes. These tiny particles are Au nanoparticles, as shown by the HRTEM image (Fig. 4(d and e)). The SAED pattern (Fig. 4(f)) reveals the crystalline nature of the Au–BO/BWO composite. Thus the microscopic studies indicate that the pristine BO/BWO composite is made up of ultrathin micron-sized flakes, and that the Au nanoparticles are decorated on these flakes in the case of the Au–BO/BWO composite. Because of the nanometric dimensions (thickness of flakes and size of Au particles), the phrase Au–BO/BWO nanocomposite is used hereafter.

The supercapacitance behaviours of the pristine BO/BWO and Au–BO/BWO nanocomposites were revealed using cyclic voltammetry (CV), galvanostatic charge–discharge (GCD), and electrochemical impedance spectroscopy (EIS) measurements performed in a typical three-electrode cell set-up. In the initial set of CV measurements, the potential window and scan rate were varied in order to maintain the shape of the CV curve, which is indicative of the capacitive behaviour of the electrode material. The CV characteristics of the BO/BWO and Au–BO/BWO electrodes were recorded at different scan rates, varying from 5 to 100 mV s^−1^, sweeping the potential from −0.2 to 0.55 V (*versus* the reference electrode, Ag/AgCl). The CV curves recorded at these scan rates are shown in Fig. S1(a and c) of the ESI.† All of the CV curves exhibit similar shapes, indicating similar redox behaviours. The oxidation and reduction peaks (Fig. S1(a)†) for BO/BWO are observed at 0.36 and 0.24 V, respectively. At the scan rate of 5 mV s^−1^, the specific capacitance value was calculated to be 96.133 F g^−1^, while it was 22.85 F g^−1^ at 100 mV s^−1^. This decrease in specific capacitance value and energy storage performance is due to the slow diffusion process at the higher scan rate (Table 1).

**Table 1:** Electrochemical performances of bismuth-based composites tested using three-electrode systems. The present work displays an enhancement in the cycling stability of BO/BWO after decoration with Au nanoparticles

Materials	Synthesis technique	Electrolyte	Specific capacitance (F g^−1^)	Stability	Reference no.
Bi_2_O_3_ nanobelts	Electrochemical deposition	1.0 M Na_2_SO_4_	250	100%	9
Bi_2_O_3_ nanowires	Vapour deposition	6 M KOH	691.3	75.5%	7
Bi_2_WO_6_	Hydrothermal	6 M KOH	574	50%	12
Bi_2_WO_6_/RGO	Hydrothermal	6 M KOH	932	100%	12
Bi_2_O_3_/Bi_2_WO_6_	Hydrothermal	1 M KOH	148.81	95.99%	Present work
Au–Bi_2_O_3_/Bi_2_WO_6_	Hydrothermal	1 M KOH	495.05	103.26%	Present work

The oxidation peak is due to the oxidation of Bi metal to Bi(iii) whereas the reduction peak is due to the reduction of Bi(iii) to Bi metal. The possible mechanisms involved during the oxidation and reduction processes are as shown below:^25^

Reduction:

1 BiO_2_^−^ → BiO_2(ads)_^−^

2 BiO_2(ads)_^−^ + e^−^ → BiO_2(ads)_^2−^

3
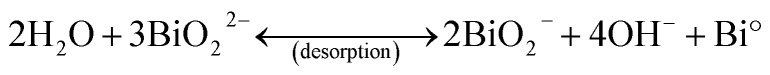

4 Bi° → Bi_(metal)_

Oxidation:

5 Bi_(metal)_ → Bi^+^ + e^−^

6
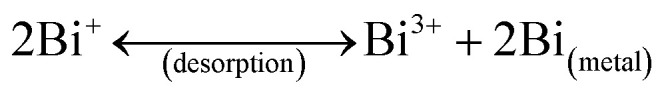

7 3OH^−^ + Bi^3+^ → Bi(OH)_3_

8 Bi(OH)_3_ → BiOOH + H_2_O

The CV curves of Au–BO/BWO recorded at different scan rates ranging from 5 to 100 mV s^−1^, in the potential window of −0.2 V to 0.5 V (*versus* Ag/AgCl) are displayed in Fig. S1(c).† The oxidation and reduction peaks for the Au–BO/BWO electrode appear at 0.37 and 0.22 V, respectively.

The GCD curves for the BO/BWO electrode were recorded at different current densities ranging from 1 to 5 A g^−1^, in the same potential window as that used for the CV measurements, and are shown as Fig. S1(b).† At the current density of 1 A g^−1^ the specific capacitance was estimated to be 148.81 F g^−1^. The specific capacitance value was observed to decrease with increasing current density. At the current density of 5 A g^−1^, its value was found to be 30.285 F g^−1^.

The GCD characteristics for the Au–BO/BWO electrode, recorded at different current densities varying from 1 to 5 A g^−1^ in the same potential window as that used for CV, are shown as Fig. S1(d).† In this case too, the specific capacitance was found to decrease with increasing current density. The values of specific capacitance, calculated at current densities of 1 and 5 A g^−1^, are 495.05 and 102.07 F g^−1^, respectively. The specific capacitance decreases at the high current density because the charges do not get enough time to migrate.

**Fig. 5:**
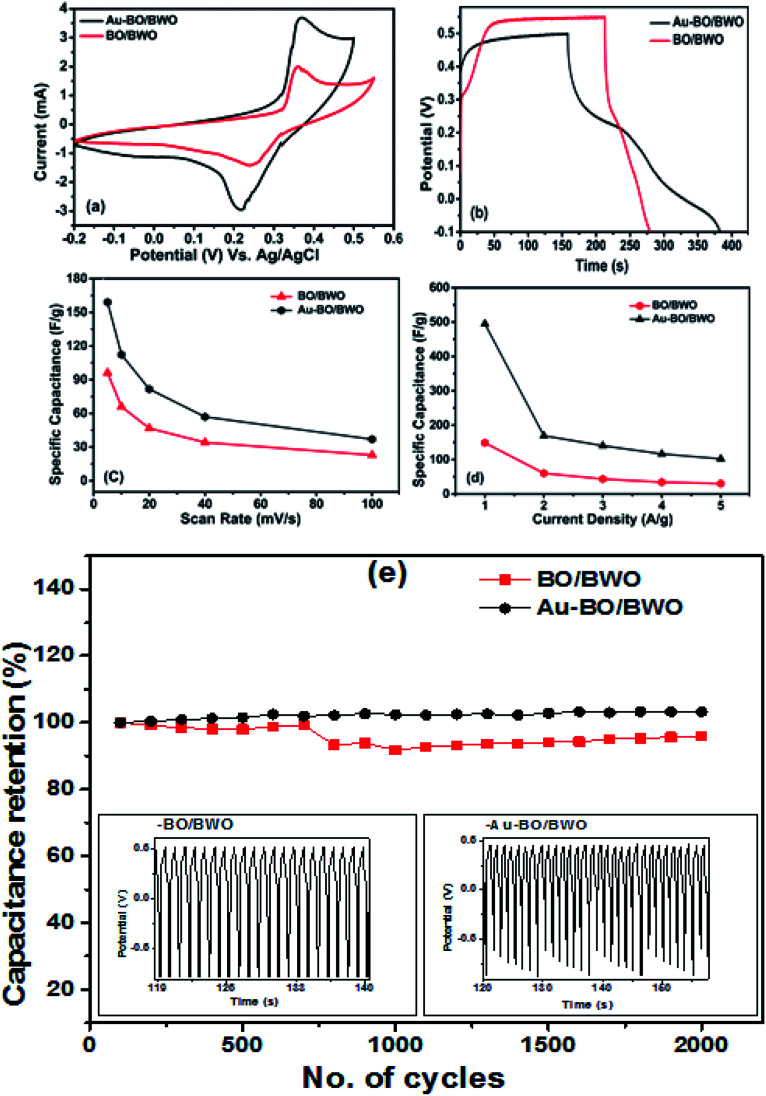
(a) CV curves of BO/BWO and Au–BO/BWO at 10 mV s^−1^ (b) GCD curves of BO/BWO and Au–BO/BWO at 1 A g^−1^ of current density. (c) Graph of specific capacitance *vs.* scan rate and (d) graph of specific capacitance *vs.* current density. (e) Cycling stability over 2000 cycles.

The optimised CV curves of pristine BO/BWO and Au–BO/BWO electrodes, recorded at a scan rate of 10 mV s^−1^, in 1 M aqueous KOH electrolyte (Fig. 5(a)), indicate that both of the electrodes show pseudocapacitive behaviour. Fig. 5(b) shows the GCD curves of both electrodes recorded at a constant current density of 1 A g^−1^. Fig. 5(c) displays the variation of specific capacitance as a function of scan rate, whereas the variation in specific capacitance with current density is depicted in Fig. 5(d). The cycling stability of both of the electrodes measured over 2000 cycles is shown in Fig. 5(e). A capacitance retention of 95.99% for BO/BWO was observed. A capacitance retention of 99.26% was observed for Au–BO/BWO, which is a gain in capacitance of about 3%, suggesting a highly reversible redox reaction for the pseudocapacitive material. It also signifies a minimum loss of energy and a good cyclic stability in comparison to those of the pristine electrode.^26^

**Fig. 6:**
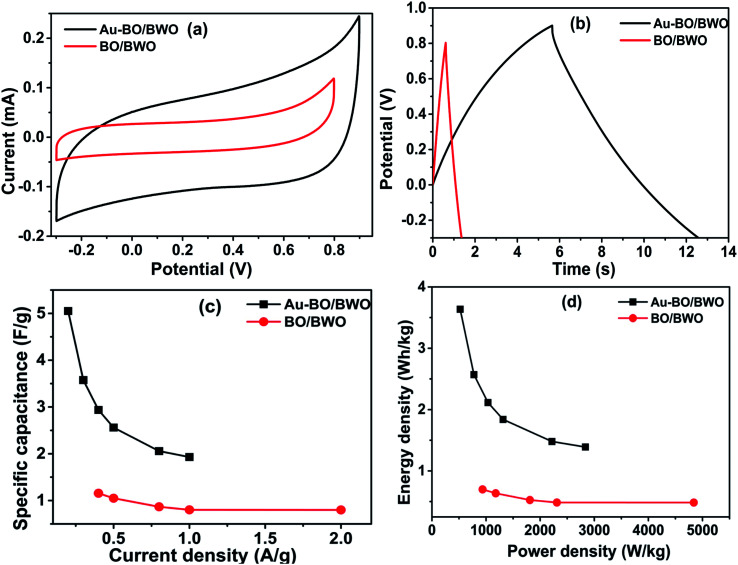
Supercapacitor electrode performance in a two-electrode configuration: (a) CV curves of BO/BWO and Au–BO/BWO at 100 mV s^−1^, (b) GCD curves of BO/BWO and Au–BO/BWO at lower current density, (c) graph of specific capacitance *vs.* current density, and (d) comparative Ragone plots.

To study the energy density and power density characteristics of both electrodes, a two-electrode symmetric assembly in a Swagelok-type cell was employed. The CV and GCD characteristics recorded at different scan rates and current densities are shown in Fig. S3(a, b) and (c, d),† respectively. Fig. 6(a) displays the CV curves of the Au–BO/BWO and BO/BWO electrodes, recorded at a scan rate of 100 mV s^−1^. The CV curves show that an enhancement of current is observed for the Au–BO/BWO electrode in comparison to the BO/BWO electrode at a fixed scan rate. This observation indicates the enhanced energy storage performance of the Au–BO/BWO electrode. The absence of redox peaks is due to the charging and discharging of the supercapacitor at a pseudo constant rate across the voltammetric cycles.^27^Fig. 6(b) shows the GCD curves recorded at lower current densities and Fig. 6(c) depicts a variation in the specific capacitance with current density. For the Au–BO/BWO, the specific capacitance calculated from the GCD curve (current density 0.2 A g^−1^) is 5.05 F g^−1^. For the BO/BWO electrode, the specific capacitance at the current density of 0.4 A g^−1^ is found to be 1.156 F g^−1^. The variation in energy and power densities, calculated using the GCD curves, is displayed in Fig. 6(d). For the pristine BO/BWO electrode, the energy density is found to be 0.699 W h kg^−1^ at 935.74 W kg^−1^, and the energy density of 0.484 W h kg^−1^ is retained at 4840 W kg^−1^. For the Au–BO/BWO electrode, the maximum energy density is found to be 3.636 W h kg^−1^ at 521.66 W kg^−1^, and an energy density of 1.389 W h kg^−1^ is retained at 2836.73 W kg^−1^. Thus, the Au–BO/BWO electrode exhibits improvements in energy density and power density, which can be attributed to the active role of the Au nanoparticles, as revealed by EIS.

To complement the EIS studies, the electrolytic properties of both of the electrodes were investigated in 0.1 M [Fe(CN)_6_]^3−/4−^ in 0.1 M KCl solution, and the CV curves are depicted in Fig. S2(a and b) of the ESI.† The Au–BO/BWO electrode exhibited an anodic peak current (*I*_pa_) of 2.32 μA, whereas the pristine BO/BWO showed 2.23 μA. The higher anodic current clearly reveals that the Au–BO/BWO electrode shows higher electrolytic activity than the BO/BWO electrode. Also, [Fe(CN)_6_]^4−^ ions partially diffuse away from the electrode before re-oxidizing so a smaller peak is obtained than expected. This indicates fast ion transfer as the redox reaction between [Fe(CN)_6_]^3−^/[Fe(CN)_6_]^4−^ is equal to the peak voltage generated by the diffusion-controlled charges.^28,29^

**Fig. 7:**
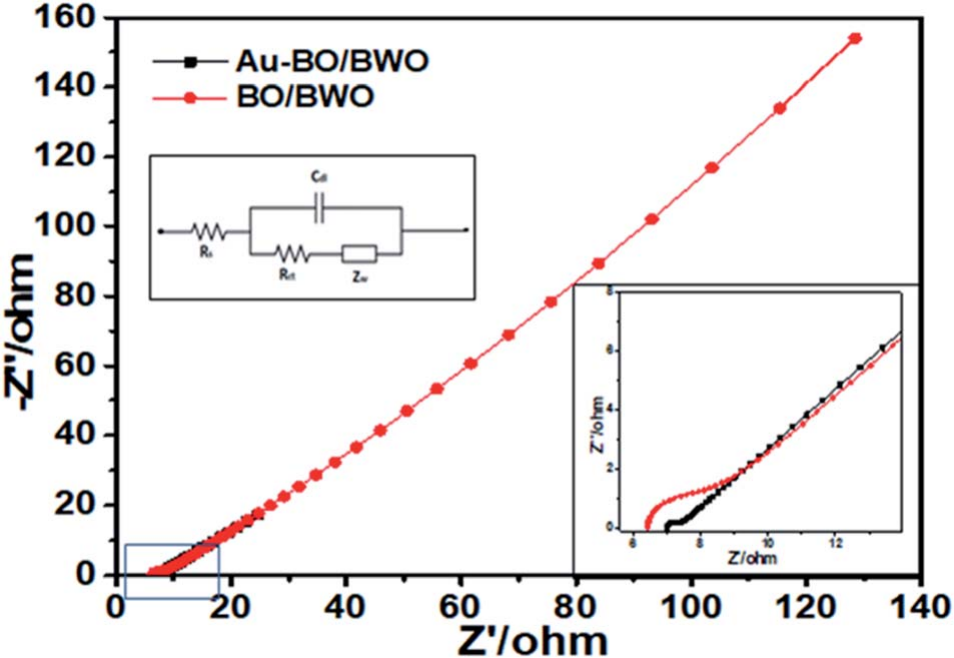
Nyquist plots of BO/BWO and Au–BO/BWO. The insets show the equivalent circuit and magnified high frequency region.

To investigate the electrochemical performances of the prepared electrodes, EIS characterization was performed. The capacitive behaviour is depicted by increase in the imaginary part of the impedance in the lower frequency region, whereas a semicircle in the high frequency region is due to charge transfer resistance of the electrode. The Nyquist plot (Fig. 7) shows a semicircular arc in the high frequency region and a straight line at 45° with respect to the real axis in the low frequency region. The diameter of the semicircle is indicative of charge transfer resistance (*R*_ct_), *i.e.*, the resistance of the contact interface between the working electrode and the electrolyte. In the present studies, the values of *R*_ct_ are estimated to be 1.53 and 0.34 Ω for the BO/BWO and Au–BWO electrodes, respectively.

The left inset of Fig. 7 displays an equivalent circuit with a double layer capacitive element (*C*_dl_) and Warburg impedance (*Z*_W_). The Warburg resistance, estimated from the slope of the straight line at 45°, is representative of the ionic charge exchange between the electrode and electrolyte. The total resistance, termed as the series resistance (*R*_s_), is a combination of electrolyte resistance, intrinsic resistance of the electrode material, and contact resistance, and is estimated from the *x*-intercept of the semicircle. The values of *R*_s_ are found to be 6.97 and 6.38 Ω for the BO/BWO and Au–BO/BWO electrodes, respectively. This asserts that the decoration of the BO/BWO nanoflakes by Au nanoparticles improves the electrical conductivity of the composite and hence leads to the rapid charge exchange between the composite and electrolyte.

## Conclusions

The nanocomposites of BO/BWO and Au–BO/BWO were synthesized *via* a hydrothermal route. Various characterization techniques (XRD, XPS, FESEM, and HRTEM) were performed for the structural and compositional analyses of the prepared materials. The electrochemical performance of the prepared electrodes was tested using three and two-electrode systems in 6 M KOH electrolyte. The Au–BO/BWO showed a specific capacitance of 495.05 F g^−1^ at 1 A g^−1^ with a good capacitance retention of 99.26% over 2000 cycles, whereas the BO/BWO showed a specific capacitance of 148.81 F g^−1^ at 1 A g^−1^. Moreover, the *R*_ct_ values calculated from the semicircles in the Nyquist plots indicated that the Au–BO/BWO electrode showed better electrical conductivity than the pristine electrode. The obtained energy density for Au–BO/BWO was 3.636 W h kg^−1^ at 521.66 W kg^−1^ which was higher than that of BO/BWO. The enhancement in BO/BWO supercapacitor performance after Au-nanoparticle decoration was due to the provision of better ionic conductivity. This investigation demonstrates that the material can be considered as an ideal material for supercapacitor electrode application.

## Conflicts of interest

There are no conflicts to declare.

## Supplementary Material

RA-009-C9RA06112F-s001
