# Supplementary material for: Development of pristine and Au-decorated Bi2O3/Bi2WO6 nanocomposites for supercapacitor electrodes
Source: RSC Adv. 2019 Oct 11;9(56):32573–80. doi: 10.1039/c9ra06112f (PMC9073182; doi:10.1039/c9ra06112f)
Supplement: RA-009-C9RA06112F-s001 [file RA-009-C9RA06112F-s001.pdf]

Electronic Supplementary Material (ESI) for RSC Advances.
This journal is © The Royal Society of Chemistry 2019

Electronic Supplementary Information

Development of pristine and Au-decorated Bi_2O_3/Bi_2WO_6 nanocomposites for supercapacitor electrode

Gorkshnath H. Gote, [1, 2] ,Mansi Pathak[3] , Mahendra A. More,[1*]Dattatray J. Late[2*], Chandra Sekhar Rout[3]

[1]Centre for Advanced Studies in Materials Science and Condensed Matter Physics, Department of Physics, SavitribaiPhule Pune University, Pune, Maharashtra 411007, India,

[2] Physical and Materials Chemistry Division, CSIR-National Chemical Laboratory, Dr. Homi Bhabha Road, Pashan, Pune, Maharashtra, 411008, India,.

[3]Centre for Nano and Material Sciences, Jain Global Campus, Jakkasandra, Ramanagaram, Bangalore - 562112, India.

1. Calculation of electrochemical properties

a. Specific capacitance calculated using cyclic voltammetry,

$$Csp = \frac{\int I(V)dv}{2[m\ s\ \Delta V]}$$

Where, m is the mass of electrode deposited on substrate, s is the scan rate, ΔV is the potential window and ∫I(V) dV represents area under the curve.

b. Specific capacitance calculated using charge-discharge cycle,

$$Csp = \frac{I\Delta t}{m\Delta V}$$

Where, I is the current, m is the mass of electrode, Δt is discharge time and ΔV is the potential window. Current/mass is given by current density i.e. A/g and discharge time/ potential window can be obtained by the slope of the discharging curve from galvanostatic charge-discharge plot (Δt/ΔV).

Energy density of symmetric cell is calculated using following formula,

$$Ed = \frac{1}{2}Cspx(\Delta V)^2$$

Where, Csp is a specific capacitance calculated from GCD and ΔV is the potential window. (Wh/kg)

Power density of symmetric cell is calculated by using following formula,

$$Pd = \frac{Ed}{\Delta t}$$

Where, Ed is the energy density and Δt is the discharging time. (W/kg)

Figure S1

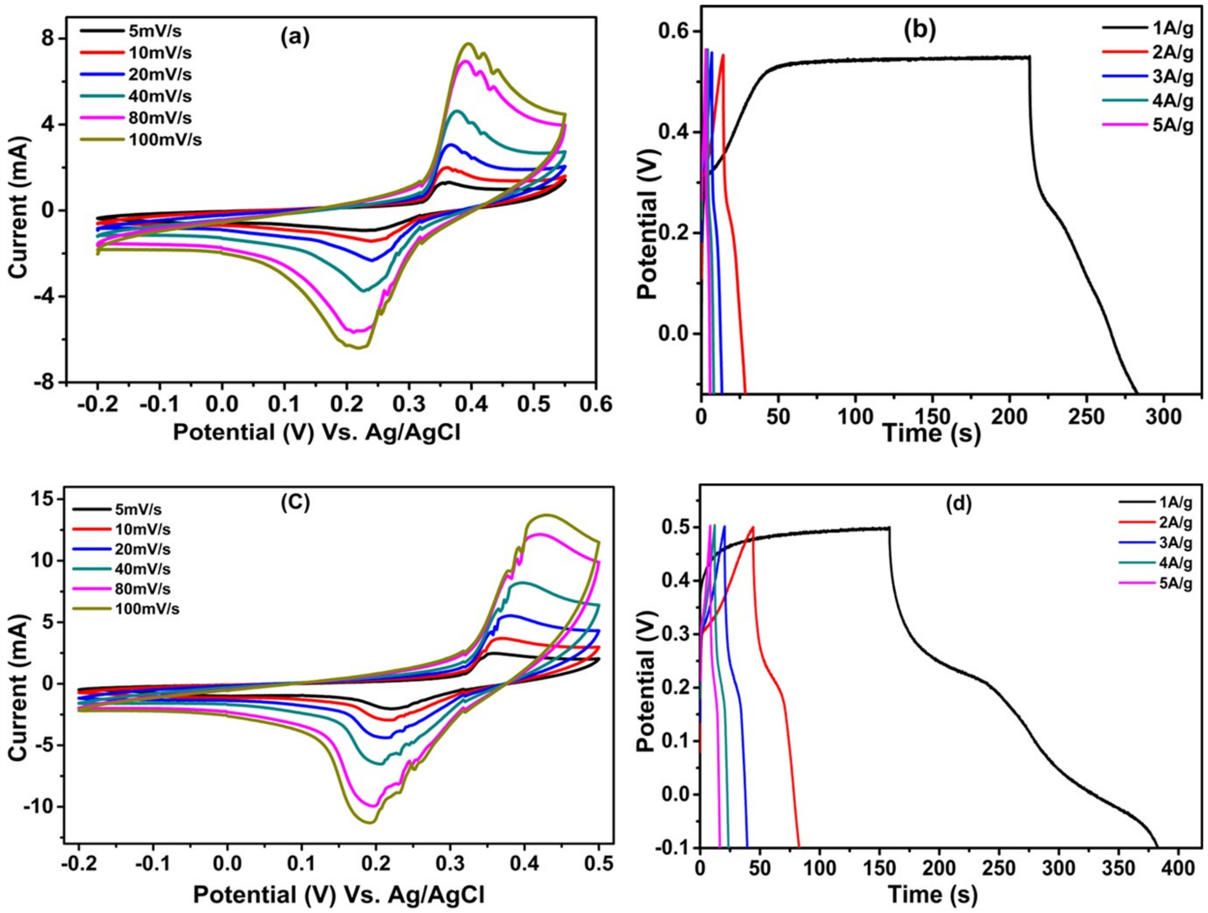

Fig. S-1(a) CV curves BO/BWO at different scan rates, (b) GCD curves of BO/BWO at different current densities, (c) CV curves of Au-BO/BWO at different scan rates and (d) GCD curves of Au-BO/BWO at different current densities.

Figure S2

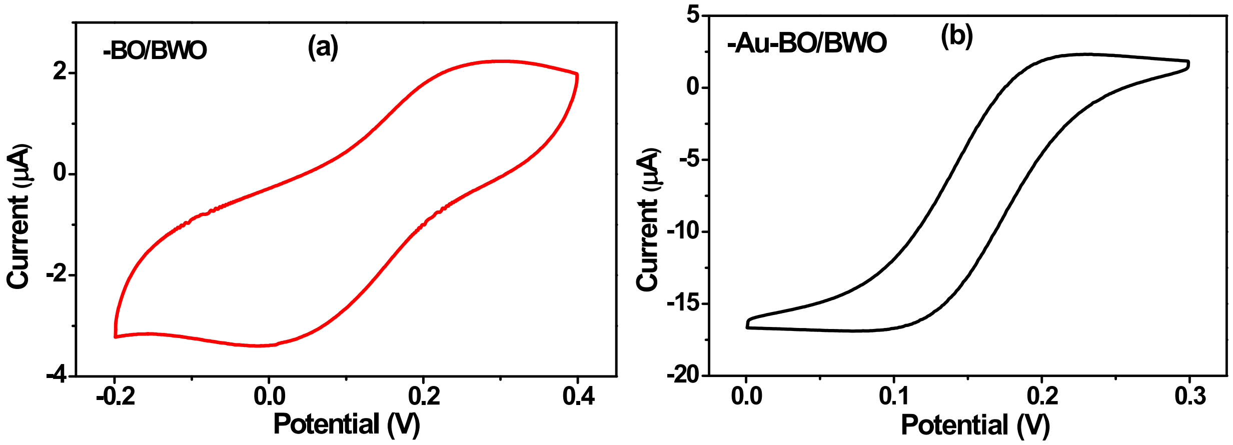

Fig S-2 CV curves of (a) BO/BWO and (b) Au-BO/BWO in 0.1 M of $[Fe(CN)_6]^{-3/-4}$ in 0.1M KCl solution.

Figure S3

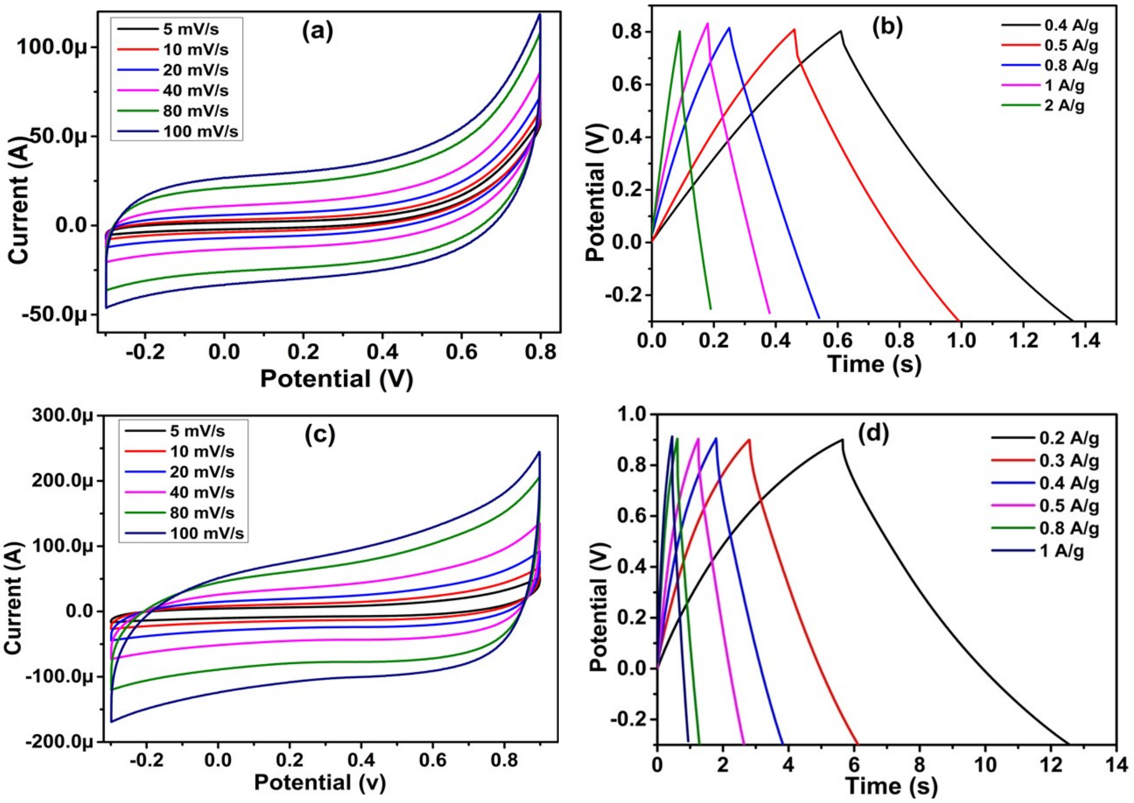

Fig S-32-electrode symmetric cell electrochemical measurements: (a) CV curves of BO/BWO for different scan rates, (b) GCD curves of BO/BWO at different current density. (c) CV curves of Au-BO/BWO for different scan rates, (b) GCD curves of Au- BO/BWO at different current density
